# Investigating RNA expression profiles altered by nicotinamide mononucleotide therapy in a chronic model of alcoholic liver disease

**DOI:** 10.1186/s40246-019-0251-1

**Published:** 2019-12-10

**Authors:** Mohammed A. Assiri, Hadi R. Ali, John O. Marentette, Youngho Yun, Juan Liu, Matthew D. Hirschey, Laura M. Saba, Peter S. Harris, Kristofer S. Fritz

**Affiliations:** 1 Department of Pharmacology and Toxicology, College of Pharmacy, King Saud University, Riyadh, Saudi Arabia; 2 Department of Pharmaceutical Sciences, Skaggs School of Pharmacy and Pharmaceutical Sciences, University of Colorado Anschutz Medical Campus, Aurora, CO 80045 USA; 3 Department of Pharmacology and Cancer Biology, Duke University Medical Center, Durham, NC 27710 USA; 4 Department of Medicine, Division of Endocrinology, Metabolism, and Nutrition, Duke University Medical Center, Durham, NC 27710 USA

**Keywords:** Alcoholic liver disease, RNA-seq, Liver, NMN, ATF3, ERK1/2, Sirtuin

## Abstract

**Background:**

Chronic alcohol consumption is a significant cause of liver disease worldwide. Several biochemical mechanisms have been linked to the initiation and progression of alcoholic liver disease (ALD) such as oxidative stress, inflammation, and metabolic dysregulation, including the disruption of NAD^+^/NADH. Indeed, an ethanol-mediated reduction in hepatic NAD^+^ levels is thought to be one factor underlying ethanol-induced steatosis, oxidative stress, steatohepatitis, insulin resistance, and inhibition of gluconeogenesis. Therefore, we applied a NAD^+^ boosting supplement to investigate alterations in the pathogenesis of early-stage ALD.

**Methods:**

To examine the impact of NAD^+^ therapy on the early stages of ALD, we utilized nicotinamide mononucleotide (NMN) at 500 mg/kg intraperitoneal injection every other day, for the duration of a Lieber-DeCarli 6-week chronic ethanol model in mice. Numerous strategies were employed to characterize the effect of NMN therapy, including the integration of RNA-seq, immunoblotting, and metabolomics analysis.

**Results:**

Our findings reveal that NMN therapy increased hepatic NAD^+^ levels, prevented an ethanol-induced increase in plasma ALT and AST, and changed the expression of 25% of the genes that were modulated by ethanol metabolism. These genes were associated with a number of pathways including the MAPK pathway. Interestingly, our analysis revealed that NMN treatment normalized Erk1/2 signaling and prevented an induction of Atf3 overexpression.

**Conclusions:**

These findings reveal previously unreported mechanisms by which NMN supplementation alters hepatic gene expression and protein pathways to impact ethanol hepatotoxicity in an early-stage murine model of ALD. Overall, our data suggest further research is needed to fully characterize treatment paradigms and biochemical implications of NAD^+^-based interventions.

## Background

Chronic alcohol consumption continues to be a significant cause of liver disease worldwide. The initiation and progression of alcoholic liver disease (ALD) arise through a complex etiology, including oxidative stress, inflammation, and metabolic dysregulation. A central factor in the disruption of hepatocyte metabolism is an ethanol-mediated reduction in NAD^+^ levels and these alterations are thought to play a critical role in ethanol-induced steatosis, oxidative stress, steatohepatitis, insulin resistance, and inhibition of gluconeogenesis [37, 42, 51, 64]. This NAD^+^ depletion occurs during the hepatic conversion of ethanol to acetaldehyde by alcohol dehydrogenase (ADH) and then to acetate by aldehyde dehydrogenase (ALDH2), as NAD^+^ is a cofactor converted to NADH by each of these enzymes [53].

NAD^+^ is an essential cofactor in vital cellular functions including redox reactions, metabolism, and DNA repair mechanisms [9]. Boosting cellular NAD^+^ levels is an emerging area of investigation for the treatment of several metabolic conditions. A growing number of reports have examined the use of NAD^+^-enhancing supplements like nicotinamide riboside (NR) and nicotinamide mononucleotide (NMN) in ameliorating obesity, aging, and diabetes [8, 65]. NMN is a precursor for NAD^+^ synthesis and nicotinamide-nucleotide adenylyl transferase (NMNAT1-3) enzymes convert NMN to NAD^+^. Several reports have demonstrated that a dose of 500 mg/kg of NMN successfully increased liver NAD^+^ levels, prevented glucose intolerance in type 2 diabetes, improved insulin sensitivity, and prevented aging-related symptoms through multiple mechanisms [20, 66, 70]. For example, in a model of diet-induced type 2 diabetes, NMN restored insulin signaling and ameliorated oxidative stress and inflammation by activating Sirtuin 1 (SIRT1) [70]. NMN also restored mitochondrial function in aged mice through a SIRT1-PGC1α-dependent mechanism [20]. In ALD, NR has been shown to prevent ethanol-induced liver damage via SIRT1-dependent mechanisms [67]. However, the impact of enhancing NAD^+^ levels through NMN supplementation in ALD remains uncharacterized, including its transcriptional impact.

A number of transcription factors have been associated with the pathogenesis of ALD [64]. Activating transcription factor 3 (Atf3) signaling has been associated with NAD^+^/NADH ratios, which represent hepatic lactate and pyruvate levels, linking Atf3 and hepatic metabolic status [30]. Atf3 is a stress-responsive transcription factor that binds to cAMP response element (CRE) and activates or represses gene expression based on its dimerization and the source of the stress [55]. In the liver, Atf3 is induced by various hepatotoxins including carbon tetrachloride and ethanol [12, 23, 64]. Hepatic overexpression of Atf3 has been reported to be detrimental, as elevated Atf3 was associated with liver damage and increased plasma alanine aminotransferase (ALT) and aspartate aminotransferase (AST), among other biomarkers [4]. Furthermore, a recent report demonstrated that increased Atf3 expression was associated with hepatic steatosis and Atf3 is one of many transcription factors that have been found to be upregulated in patients with alcoholic steatohepatitis [30, 50]. Additionally, Atf3 overexpression was observed in ethanol-treated human primary hepatocytes and HepG2 cells [43]. These findings provide a strong link for Atf3 in the mechanisms of ethanol-induced steatosis and oxidative stress of ALD.

In addition to hepatic signaling through Atf3, the mitogen-activated protein kinases (MAPK) are a fundamental cellular pathway that can transduce the activation of extracellular receptors to stressors like ethanol toxicity, impacting proliferation, differentiation, stress response, apoptosis, and survival [49]. Contradictory findings exist regarding the role of the MAPK pathway in ALD depending on the model of ethanol feeding [5, 35, 36]. Extracellular signal-regulated kinase1/2 (Erk1/2) is a central protein in the MAPK pathway and plays a primary role in metabolism, cell cycle, and cell survival [56]. Several reports have indicated that Erk1/2 phosphorylation is decreased in the liver due to ethanol metabolism, but results have been shown to vary depending on the model of ethanol consumption employed [5, 13, 54, 58].

In this study, we sought to evaluate the effect of ethanol metabolism and NAD^+^ supplementation on the underlying mechanisms of early-stage alcoholic liver disease. NMN supplementation in a Lieber-DeCarli 6-week chronic ethanol model was examined utilizing several approaches, including the integration of RNA-seq, immunoblotting, and metabolomics analysis. Our findings reveal that NMN therapy increased hepatic NAD^+^ levels, limited liver damage as indicated by plasma ALT and AST, and changed the expression of 25% of the genes that were modulated by ethanol metabolism. These genes were associated with a number of pathways including the MAPK pathway. Our analysis further revealed that NMN treatment restored ethanol-perturbed Erk1/2 signaling and prevented an induction of Atf3 overexpression.

## Results

### NMN prevents ethanol-induced elevation of plasma ALT and AST

**Fig. 1:**
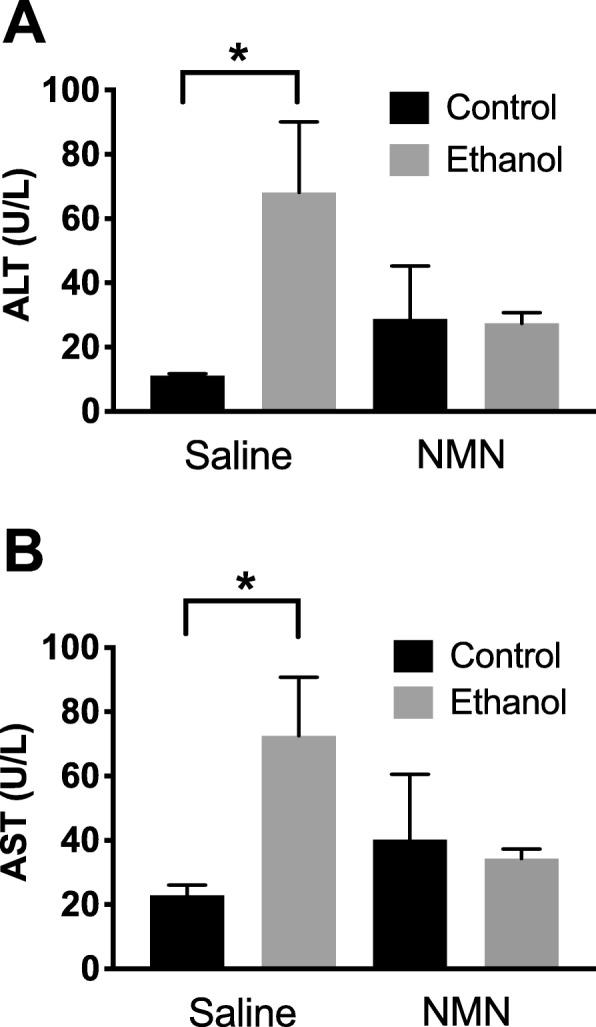
Differential ethanol effects on alanine aminotransferase (ALT) and aspartate aminotransferase (AST). **a** Treatment with NMN prevents an ethanol-induced increase in plasma ALT. **b** NMN treatment prevents an ethanol-mediated increase in AST plasma levels (mean +/− SEM) (*n* = 4 to 5 per treatment combination) (*p < 0.05)

Plasma ALT and AST are both well-characterized liver damage biomarkers. To assess whether NMN protects the liver against ethanol-induced damage, we measured plasma ALT and AST. As shown in Fig. 1, ethanol feeding in the saline group significantly increased plasma levels for both ALT (*p* = 0.020) and AST (*p* = 0.017). For both ALT and AST, the interaction effect between NMN treatment and ethanol treatment was statistically suggestive (*p* = 0.052 for ALT; *p* = 0.062 for AST), which indicates that the effect of ethanol differed between animals treated with NMN and animals treated with saline. Interestingly, NMN treatment prevented a significant ethanol-induced increase in plasma levels of both markers. These results suggest a protective role for NMN against ethanol-induced hepatic damage. However, the NMN treatment did not alter the effect of ethanol on other liver markers such as liver triglycerides, liver to body weight ratio, and plasma ethanol concentrations (*p* > 0.10 for all three; Additional file 1: Figure S1). This may be a consequence of using an early-stage model of ALD, limiting the complete development and protection of liver disease. Other confounding factors that may impact outcomes include the frequency, dose, and route of NMN administration.

### NMN alters hepatoprotective metabolites

**Fig. 2:**
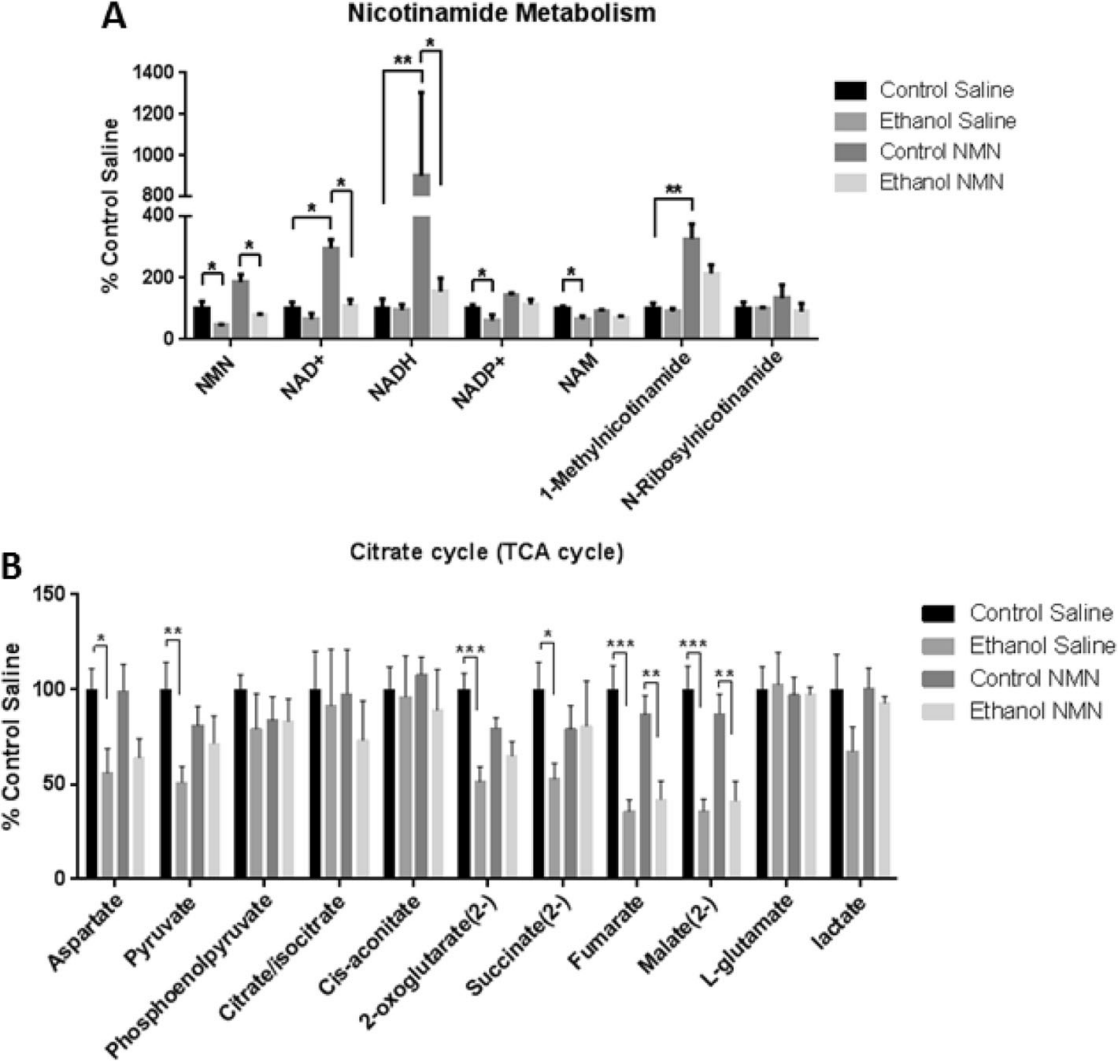
Metabolomics profile of hepatic tissue assessing the impact of NMN therapy in a model of ethanol toxicity. **a** NMN treatment significantly alters metabolites of nicotinamide metabolism. **b** TCA cycle metabolites are moderately altered due to NMN supplementation (mean +/− SEM) (*n* ≥ 4) (*p < 0.05, **p < 0.01, ***p < 0.001)

To identify the effect of ethanol with and without NMN treatment on hepatic metabolism, we assessed the polar metabolite fraction of liver tissue from Control Saline, Ethanol Saline, Control NMN, and Ethanol NMN groups. Chronic ethanol intake induces liver damage partially by depletion of NAD^+^. To confirm that the intraperitoneal (i.p.) injection of 500 mg/kg NMN increases liver concentrations of NAD^+^ and its related metabolites, we evaluated the liver content of NAD^+^, NMN, NAM, NADH, and among other NAD^+^-related metabolites. We found that ethanol feeding with NMN injections successfully maintained NAD^+^ and the majority of its metabolite levels to that of the Control Saline group (Fig. 2a). Previous work has demonstrated a significant decrease of NAD^+^ in this ALD model [18]. We also evaluated metabolites related to TCA cycle, which are known to be altered during ethanol metabolism [10]. Our results show that NMN treatment successfully prevented the effect of ethanol-induced decrease on certain TCA cycle metabolites, such as pyruvate and 2-oxoglutarate, key metabolic intermediates regulating central carbon metabolism which are known to impact hepatic function, oxidative stress, and steatosis (Fig. 2b) [17, 45]. The values of the remaining metabolites are listed in Additional file 2: Table S1. Our findings here demonstrate that the i.p. injection of NMN at 500 mg/kg every other day successfully increased NAD^+^ and its related metabolites during ethanol toxicity and suggests that NMN alters a number of key regulatory metabolites.

### RNA-seq analysis

**Fig. 3:**
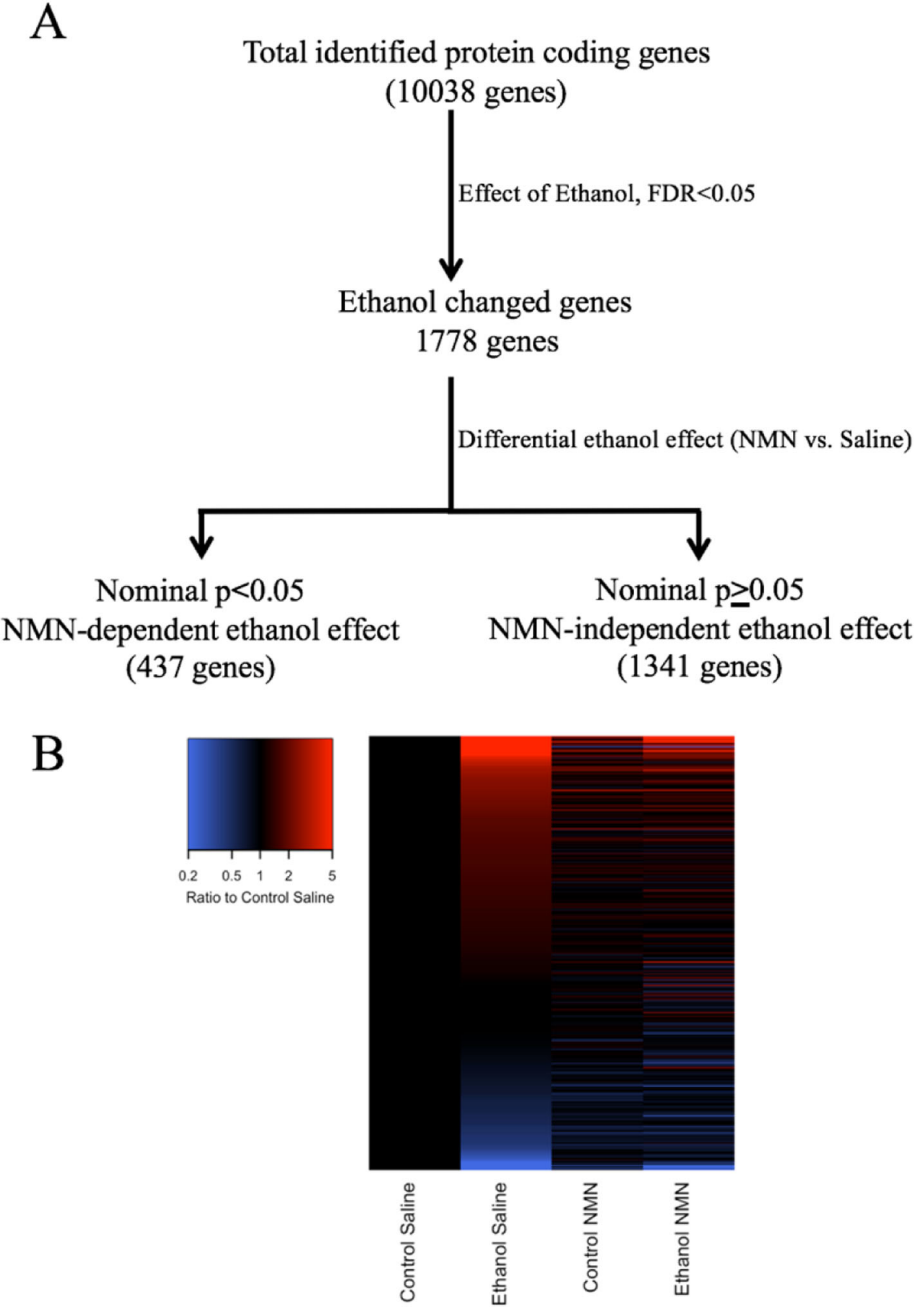
Ethanol effects on RNA expression levels via RNA sequencing analysis. **a** Pipeline for identification of NMN-dependent and NMN-independent ethanol effects on RNA expression levels. **b** RNA expression differences across the treatment (NMN and ethanol) combinations for genes with NMN-dependent ethanol effects. Each row represents a gene with a significant ethanol effect (FDR < 0.05) that was classified as NMN-dependent (differential ethanol *p* < 0.05; 437 genes). Expression levels are shown as the ratio of average expression level in each treatment combination (columns) to the average expression in the Control Saline group. Expression levels were log base 2 transformed prior to analyses and graphing. Values in the key have be back transformed to increase interpretability

We utilized next generation RNA-seq analysis by Illumina NovaSeq to comprehensively delineate the molecular mechanisms underlying how NMN alters ethanol-induced liver damage. We filtered the protein-coding genes with expression levels above background in our samples (10,038 genes) by the overall chronic ethanol effect false discovery rate (FDR) threshold of 0.05. This revealed that ethanol significantly alters the expression of 1778 genes. To identify the role of NMN in modifying the effect of ethanol on these 1778 genes, we separated these genes into two groups based on their interaction effect (NMN and ethanol; nominal *p* < 0.05). We termed the first group as NMN-dependent ethanol genes, which contains 437 genes. The second group focused on NMN-independent ethanol genes and contained 1341 genes. The genes and their categories are found in Additional file 3: Table S2. Overall, NMN treatment altered the effect of ethanol on 25% of the genes with a significant ethanol effect (Fig. 3a). Of the 437 NMN-dependent ethanol genes, most increased expression with ethanol treatment in the saline group, but that ethanol-induced increase in expression was either completely diminished in the NMN-treated group or the magnitude of the ethanol effects was reduced (Fig. 3b). Furthermore, NMN-dependent ethanol genes that were both dramatically influenced by ethanol (more than a 10-fold increase or decrease in expression) and indicated a statistically robust difference in ethanol effects among the NMN-treated and saline-treated animals (interaction *p* value < 0.0001) are shown in Table 1.

**Table 1:** Genes from the RNA-seq dataset that have an ethanol effect 10-fold or greater (FDR < 0.05) and a differential effect of ethanol of (*p* < 0.0001)

Gene symbol	Gene description	Overall effect of ethanol FDR (unadjusted *p* value)	Differential effect of ethanol in NMN and saline groups FDR (unadjusted *p* value)	Ethanol effect in saline group (ethanol expression as percent of control expression)	Ethanol effect in NMN group (ethanol expression as percent of control expression)
Hmox1	Heme oxygenase 1	3.6e−135 (2.6e−139)	3.6e-20 (3.4e−24)	1902%	328%
Nr4a1	Nuclear receptor subfamily 4	4.9e−26 (4.9e−29)	1.0e-06 (2.9e−10)	1057%	123%
Trib3	Tribbles pseudokinase 3	3.2e−54 (9.2e−58)	4.0e-06 (1.5e−09)	1483%	281%
Atf3	Activating transcription factor 3	2.6e−23 (3.2e−26)	1.7e-02 (3.9e−05)	3711%	522%
Clec2h	C-type lectin domain family 2	1.3e−10 (1.0e−12)	1.9e-02 (5.2e−05)	9%	69%

**Fig. 4:**
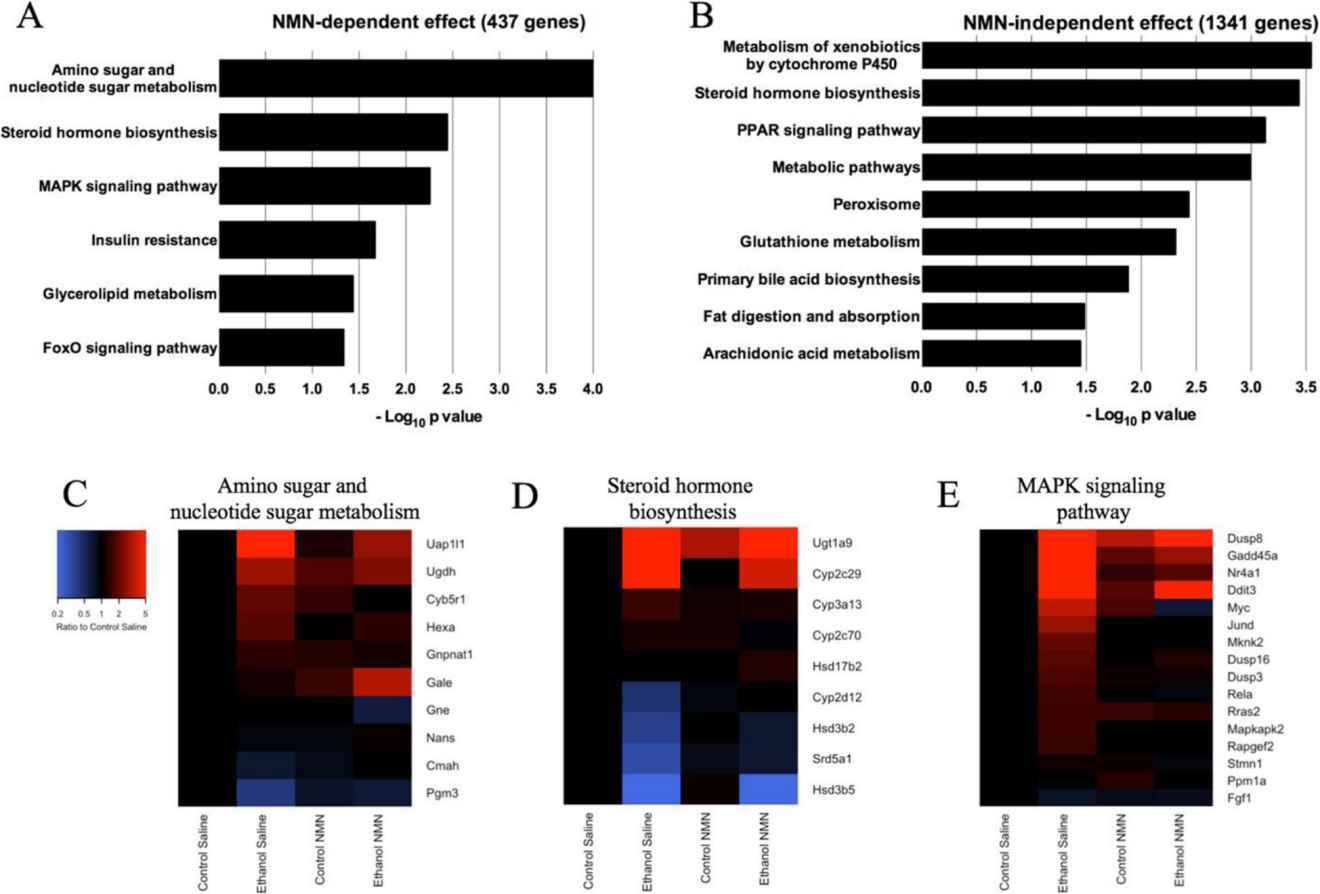
Pathway analysis and enrichment (*p* < 0.05) of **a** NMN-dependent ethanol genes and **b** NMN-independent ethanol genes. The top three (via *p* value) pathways enriched for genes with NMN-dependent ethanol effects: **c** amino sugar and nucleotide sugar metabolism, **d** steroid hormone biosynthesis, and **e** MAPK signaling pathway. In these heatmaps, each row represents a gene with a significant ethanol effect (FDR < 0.05) that was classified as NMN-dependent (differential ethanol *p* < 0.05). Expression levels are shown as the ratio of average expression level in each treatment combination (columns) to the average expression in the Control Saline group. Expression levels were log base 2 transformed prior to analyses and graphing. Values in the key have be back transformed to increase interpretability

To examine the enriched pathways associated with both ethanol sensitive groups, NMN-dependent and NMN-independent, we utilized KEGG pathway analysis. We found that NMN-dependent ethanol genes are associated with numerous pathways, including amino sugar and nucleotide sugar metabolism and MAPK signaling pathway (Fig. 4a). The expression changes of the genes from our dataset in these KEGG pathways are presented in Fig. 4c–e. These findings suggest that NMN directly impacts upon these pathways and may provide support toward protection against ethanol-induced metabolic and proteomic dysregulation. NMN-independent ethanol genes are enriched in pathways that are well-known to be affected by ethanol metabolism such as metabolism of xenobiotics by cytochrome P450, PPAR signaling pathway, peroxisome, glutathione metabolism, primary bile acid biosynthesis, fat digestion and absorption, and arachidonic acid metabolism (Fig. 4b) [21, 53].

### Baseline effect of NMN on gene expression

Since NAD^+^ is a major cellular cofactor, we assessed the effect of NMN in the control diet alone. We first filtered protein-coding genes expressed above background by NMN FDR *p* < 0.05, and then identified the upregulated and downregulated genes by comparing the gene expression between control diet with and without NMN treatment. As illustrated in Additional file 1: Figure S2, NMN significantly affected 1046 genes, as it upregulated 467 genes and downregulated 579 genes. From the KEGG pathway analysis, we found that the upregulated genes were associated with metabolic pathways, steroid biosynthesis, amino sugar and nucleotide sugar metabolism, TCA cycle, peroxisome, protein processing in endoplasmic reticulum, MAPK signaling pathway, RNA transport, and lysosome (Additional file 1: Figure S2B). On the other hand, downregulated genes were enriched in drug metabolism, MAPK signaling pathway, lysosome, glycosaminoglycan degradation, transcriptional dysregulation in cancer, PI3K-Akt signaling pathway, and metabolic pathways (Additional file 1: Figure S2C).

### Immunoblot verification of RNA-seq identified targets

**Fig. 5:**
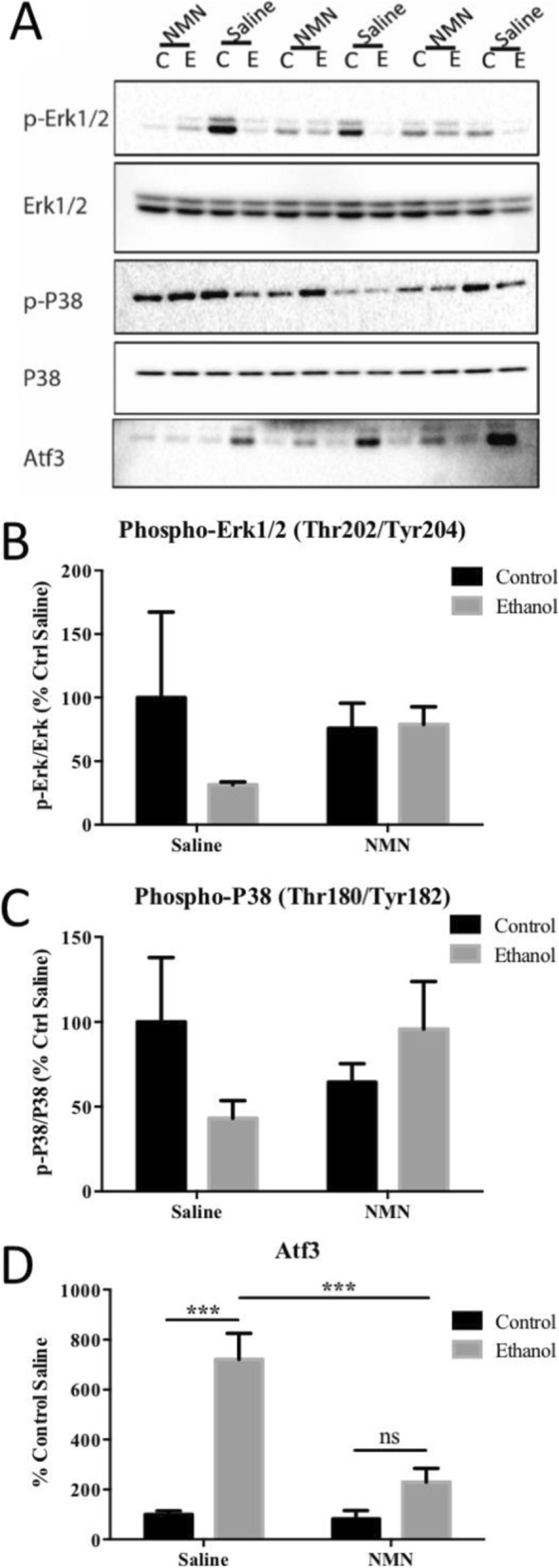
Effect of NMN treatment on Erk, P38, and Atf3. **a** Western blot image of phosho-Erk1/2, Erk1/2, phospho-P38, P38, and Atf3. **b** Densitometric analysis of phospho-Erk1/2 immunoblot normalized to total Erk1/2. **c** Densitometric analysis of phosphorylated P38 normalized to total P38. **d** Densitometric analysis of Atf3 western blot (mean +/− SEM) (***p < 0.001)

The MAPK pathway was significantly affected by NMN as shown by the RNA-seq data. In the absence of NMN, ethanol consumption significantly increased the expression of dual specificity phosphatase 8 (Dusp8) and growth arrest and DNA-damage-inducible 45 alpha (Gadd45a). Interestingly, NMN treatment altered that difference (Fig. 4e). Dusp8 and Gadd45a are both associated with regulating the phosphorylation of P38 and Erk1/2 [38, 57, 63]. Therefore, we evaluated the phosphorylation of P38 and Erk1/2 by western blot (Fig. 5). Our findings demonstrate that ethanol feeding decreased the phosphorylation of Erk1/2 and NMN prevented the inhibitory effect of ethanol on Erk1/2 phosphorylation (Fig. 5b). Also, we found that P38 phosphorylation is inhibited by ethanol in the absence of NMN and that NMN partially restored P38 phosphorylation (Fig. 5c). In aggregate, these results suggest that NMN directly affects P38 and Erk1/2 through MAPK signaling.

A key factor in metabolic stress, Atf3 is a stress-inducible gene linked to symptoms of hepatic dysfunction [4]. The expression of Atf3 was one of the top five genes with the most dramatic ethanol influence and of the most statistically robust NMN-dependent genes (Table 1). RNA-seq analysis revealed that ethanol significantly increased Atf3 gene expression and NMN ameliorated that effect. We further assessed the effect of ethanol in the presence or absence of NMN on Atf3 protein abundance by immunoblot. In agreement with the RNA-seq data, we found that ethanol significantly upregulated Atf3 and that NMN significantly prevented that effect (Fig. 5d). Our findings here demonstrate that the prevention of Atf3 overexpression by NMN is an important mechanism that may support hepatoprotection and will be a focus of follow-up studies.

## Discussion

The overarching goal of this study was to examine the impact of NMN on the pathogenesis of early-stage ALD. Supplementing NAD^+^ through NMN treatment effectively prevented ethanol-induced increases in ALT and AST plasma levels and restored Erk1/2 signaling, which was disrupted by ethanol metabolism. A key finding of this study is that NMN therapy significantly reduces Atf3, which has been linked with hepatic steatosis, oxidative stress, mitochondrial function, and ethanol-induced metabolic syndrome [29, 30]. Given the central role of NAD^+^ in hepatic metabolism, it is not surprising to find that NMN alters Erk1/2 and Atf3 signaling. Importantly, this is the first reported evidence that NMN has the potential to mitigate ethanol-induced liver injury, in part, through MAPK signaling targeted at Erk1/2 and Atf3.

Several reports have demonstrated varied physiological outcomes by elevating NAD^+^ levels through genetic mechanisms. For instance, deletion or inhibition of non-Sirtuin NAD^+^ consuming enzymes like poly(ADP-ribose) polymerase 1 (PARP1) [6] or cluster of differentiation 38 (CD38) [1] enhances cellular contents of NAD^+^. Currently, PARP1 inhibitors like olaparib, niraparib, and rucaparib are used to treat a number of malignancies including ovarian cancer [16]. Another study demonstrated the importance of maintaining NAD^+^ levels to prevent neuronal cell death by PARP inhibition [2]. Additionally, a pro-degenerative effect of accumulating NMN in axons in vivo has been observed [14].

The administration of NAD^+^ precursors like NR and NMN increases cellular NAD^+^ concentrations and has also been shown to ameliorate several pathological conditions through complex metabolic signaling mechanisms, including liver fibrosis [52]. For example, NMN treatment prevented high fat diet-induced metabolic disruption in a model of maternal obesity [65]. NMN treatment also showed therapeutic promise in alleviating aging-associated symptoms [48]. Furthermore, in a model of cardiomyopathy, NMN treatment restored cardiac function by activating SIRT3 [46]. NMN also mitigated diabetes-induced side effects including glucose intolerance and insulin signaling [70]. Preliminary research has also demonstrated positive results toward NMN therapy in ameliorating obesity-associated systemic metabolic derangements [62] and a clinical trial is currently active with the aim of better understanding the effect of NMN on metabolic health (ClinicalTrials.gov identifier: NCT03151239). While metabolically targeted NAD^+^ therapies appear promising, our results suggest the need to continue evaluating the efficacy and mechanisms of these therapies in NAD^+^-dependent pathologies like ALD, aging, and diabetes [33]. Indeed, a recent clinical trial in aged men utilized 1 g NR per day for 21 days. The authors employed muscle RNA sequencing to reveal NR-mediated downregulation of energy metabolism and mitochondrial pathways, without altering mitochondrial bioenergetics. Importantly, NR therapy did augment the skeletal muscle NAD^+^ metabolome while reducing levels of circulating inflammatory cytokines [15]. These reports suggest a potential role for NMN and NR therapy in alleviating several pathological conditions.

In this report, we employed RNA-seq to reveal novel mechanisms by which NMN alters ethanol-induced hepatic metabolism, partially by restoring Erk1/2 signaling and by the prevention of ethanol-induced Atf3 overexpression. Our findings are consistent with previous reports linking the inhibition of Erk and P38 pathways to oxidative stress, cholesterol metabolism, and liver regeneration [19, 26, 58, 68]. Since NMN rescued the inhibition of Erk1/2, which is involved in liver regeneration, we anticipate NMN could be a candidate therapeutic option for situations where Erk1/2 signaling is disrupted. Caution should be used when approaching a metabolic intervention such as NAD^+^-based therapy, since it directly alters the NAD^+^/NADH redox state of numerous organs and cell types and impacts a host of NAD^+^-dependent proteins and pathways. Furthermore, questions remain regarding appropriate doses, frequency of treatment, and routes of administration. Alternatively, several reports revealed that ethanol activates the Erk signaling pathway by lipopolysaccharide (LPS) and acetaldehyde-dependent mechanisms [11, 44]. The evidence presented herein suggests further research is needed to fully characterize the efficacy of NMN therapy in situations of ALD.

Atf3 is a stress-response transcription factor that activates or suppresses the expression of several genes depending on its dimerization, cell type, and stressor [23]. Importantly, Atf3 overexpression is a known detrimental factor in liver damage, as it is linked to increasing plasma levels of ALT and AST [4]. Ethanol-induced Atf3 overexpression is implicated in ethanol-mediated inhibition of gluconeogenesis [64]. Consistent with these findings, our metabolomics results show that in the absence of NMN, ethanol metabolism significantly depleted pyruvate levels and that effect was mitigated by NMN treatment. A recent report demonstrated that Atf3 played a key role in oxidative stress-mediated hepatic steatosis and that Atf3 silencing reduced ALT levels [30]. Their findings directly linked Atf3 to NAD^+^/NADH ratios and pyruvate, which support our findings regarding how NMN therapy impacts ethanol-induced hepatotoxicity. Since NMN treatment mitigates Atf3 overexpression, future research is needed to evaluate the role of NMN in treating other conditions that are mediated by Atf3 overexpression, such as hepatic ischemia [22].

In addition to pyruvate, our metabolomics analysis identified 2-oxoglutarate as a metabolite of interest that is recovered due to NMN therapy. While ethanol metabolism significantly decreased 2-oxoglutarate, NMN intervention prevented this decrease. The underlying mechanism for this change remains unknown; however, 2-oxoglutarate is a key metabolite related to mitochondrial function and health [69]. Previously published results demonstrate that NMN improves mitochondrial function, and this result may be an indicator of such outcome [25, 60, 61].

Mitochondrial protein hyperacetylation is a key consequence of ethanol metabolism [3, 59]. Sirtuins are a group of NAD^+^-dependent deacetylase enzymes responsible for removing protein acetylation, and SIRT3 is the main mitochondrial deacetylase. Since NMN treatment increases NAD^+^ levels, we evaluated the effect of NMN therapy on mitochondrial protein acetylation, including the known SIRT3 target lysines of SOD2. Regardless of NMN treatment, ethanol significantly increases protein acetylation throughout the cell (Additional file 1: Figures S3 and S4). These findings are contrary to other reports that showed NMN and NR treatment activates SIRT3 to deacetylate mitochondrial protein [31, 67]. These differences may be due to the treatment paradigm, such as dose, timing, route of administration, and duration. Further research is necessary to critically evaluate the effect of NMN treatment on sirtuin activity, in the context of ALD and other metabolic disorders.

## Conclusion

In conclusion, our findings demonstrate that NMN supplementation restored NAD^+^ levels in ethanol-fed mice to levels seen in the control group, which prevented ethanol-induced elevation of ALT and AST, in part, through Atf3 and Erk1/2 signaling. Other measures, such as hepatic triglycerides and liver to body weight ratio, showed no changes due to NMN treatment. Utilizing a more severe model of ALD may reveal a significant recovery of other liver pathologies, which may not be clearly defined in our early-stage model of ethanol toxicity. Clearly, further research is needed to investigate how NAD^+^ therapies impact the pathogenesis of ALD, including comparing biochemical mechanisms of NR and NMN in recovering hepatocyte function and health.

## Materials and methods

### Animal study design

All procedures involving animals were approved by the Institutional Animal Care and Use Committee of the University of Colorado and were performed in accordance with the published National Institutes of Health guidelines. C57BL/6 J mice were obtained from Jackson Laboratories at the age of 7 weeks and then kept for a week for acclimation before initiating the study. A 6-week Lieber-DeCarli chronic ethanol model was applied (ethanol catalog #S4474SP and control catalog #S4473SP, Bio-Serv, Frenchtown, NJ). The diets consisted of 44% fat-derived calories, 16% protein-derived calories and the remaining balance being comprised of either carbohydrate or ethanol-derived calories (EDC). Ethanol-fed mice started the study on a diet consisting of 2% ethanol (v/v), with ethanol-derived calories (EDC) increased on a weekly basis until sacrifice; week 6 consisted of 6% ethanol (v/v) or 31.8% EDC. Pair-fed animals were calorically matched to an ethanol-fed mouse where EDC were replaced with maltodextrin. Twenty male C57BL/6 J mice were divided into two major groups: 10 mice in group A treated with NMN at a dose of 500 mg/kg every other day, prior to evening feedings, and 10 mice in group B injected with normal saline as a control group for the i.p. injection. Each group (groups A and B) was divided into two subgroups, one fed control diet (five mice) and the second group fed ethanol-containing diet (five mice) resulting in four subgroups: Control Saline, Ethanol Saline, Control NMN, and Ethanol NMN. All 20 male mice (8 weeks old) were fed a modified Lieber-DeCarli liquid-based diet for 6 weeks, with a fresh diet provided between 3 p.m. and 5 p.m. daily. Upon completion of the study, animals were anesthetized via intraperitoneal injection of sodium pentobarbital and euthanized via exsanguination. Livers were excised, weighed, and frozen for biochemical characterization, or subjected to differential centrifugation using a sucrose buffer for mitochondrial and cytosolic subcellular fractionation as previously described [24]. Measurement of ALT, AST, and triglycerides was performed according to Sekisui Diagnostics kits protocol: ALT (#318-30), AST (#319-30), and triglycerides (#236-60) (SEKISUI Diagnostics Lexington, MA, USA).

A heterogeneous covariance model was used for statistical analyses of ALT, AST, liver triglycerides, liver weight (compared to body weight), and plasma ethanol levels. Treatment with NMN (NMN vs. saline), exposure to ethanol (ethanol vs. control), and their interaction were used as independent variables within a linear regression model that allowed the within-group variance to differ between groups (group = the combination of NMN and ethanol treatments). Specific comparisons between groups were estimated using linear contrasts. These models were executed using the MIXED procedure in SAS (version 9.4; SAS Institute Inc., Cary, NC, USA). Figures were generated using GraphPad Prism 6.04 (GraphPad, La Jolla, CA, USA).

### Metabolomics analysis

Liver metabolites were extracted by homogenization of 5–10 mg of liver tissue with methanol, MTBE, and H_2_O as previously described [39]. The final extraction solvent composition was methanol/MTBE/H_2_O (1/3/1). Samples were centrifuged at 20,000 rcf at 4 °C for 10 min. Samples were split into two formed layers, polar and non-polar metabolites, into two tubes, and the bottom polar metabolite layer was speed-vacuum dried and stored at − 80 °C. The amount of metabolite analyzed was equal to 2 mg of tissue.

An ultimate 3000 UHPLC (Dionex) was coupled to a Q Exactive Plus-Mass spectrometer (QE-MS, Thermo Scientific) for metabolite profiling. A hydrophilic interaction chromatography (HILIC) methodemploying an Xbridge amide column (100 × 2.1 mm i.d., 3.5 μm; Waters) was used for polar metabolite separation. Detailed LC method was described previously [40] except that mobile phase A was replaced with water containing 5 mM ammonium acetate (pH 6.8). The QE-MS was equipped with a HESI probe with related parameters set as below: heater temperature, 120 °C; sheath gas, 30; auxiliary gas, 10; sweep gas, 3; spray voltage, 3.0 kV for the positive mode and 2.5 kV for the negative mode; capillary temperature, 320 °C; S-lens, 55; scan range (*m/z*), 70 to 900 for positive mode (1.31 to 12.5 min) and negative mode (1.31 to 6.6 min) and 100 to 1000 for negative mode (6.61 to 12.5 min); and resolution, 70,000; automated gain control (AGC), 3 × 10^6^ ions. Customized mass calibration was performed before data acquisition.

LC-MS peak extraction and integration were performed using commercially available software Sieve 2.2 (Thermo Scientific). The peak area was used to represent the relative abundance of each metabolite in different samples. The missing values were handled as described previously [40]. Intensity values were normalized to the average saline control and these ratios were log base 2 transformed for statistical analyses. Ethanol effects and differential ethanol effects were tested using a two-way ANOVA model. This model included main effects for chronic ethanol exposure and NMN exposure and an interaction effect between these two factors. Chronic ethanol effects were identified using an F statistic and the “ANOVA” function in R to test the hypothesis that there is a significant main effect of ethanol and/or a significant interaction between ethanol and NMN treatments. Differential ethanol effects, i.e., the effect of chronic ethanol differed between the group of animals exposed to NMN and the group of animals that were not, were also identified using an F statistic from the analysis of variance table to test the hypothesis that there is a significant interaction between ethanol and NMN. Two groups of metabolites were identified: (1) NMN-dependent ethanol metabolites and (2) NMN-independent ethanol metabolites. A false discovery rate (FDR) [7] was used to adjust the overall ethanol effect for multiple testing. Graphics were generated using R and using Prism 6.04 (GraphPad, La Jolla, CA, USA).

### RNA sequencing

Total RNA was isolated and purified from mouse liver (*n* = 3 per group) according to the RNeasy Plus Mini Kit (QIAGEN, Hilden, Germany). RNA quality and concentrations were determined by NanoDrop 2000 spectrophotometer (Thermo Scientific, Waltham, MA, USA). RNA libraries were constructed using the Illumina TruSeq Stranded mRNA Library Prep Kit (San Diego, CA, USA) in accordance with the manufacturer’s protocol. Part of this process included isolation of polyadenylated transcripts via Oligo-dT beads that capture polyA tails. An Agilent Technologies Bioanalyzer 2100 was utilized to assess sequencing library quality, and samples were sequenced 2 × 150 bp paired-end reads on an Illumina NovaSeq at the University of Colorado Genomics Core.

Raw reads were trimmed to remove adapter sequences as well as low-quality bases using cutadapt (v. 1.9.1) [47]. Low-quality bases were determined using the default parameters. The trimmed reads were aligned to the Ensembl mouse transcriptome (GRCm38.93) and gene-level estimates of abundance were derived using the RSEM (RNA-Seq by Expectation Maximization) algorithm (v. 1.2.31) [34]. Genes were included in further statistical analyses if at least 50% of the samples had 10 or more reads. To identify ethanol effects, we used a negative binomial regression model with an empirical Bayes shrinkage of the dispersion parameter in the DESeq2 package (v. 1.20.0) [41]. This model included main effects for chronic ethanol exposure and NMN exposure and an interaction effect between these two factors. Chronic ethanol effects were identified using a likelihood ratio statistic to test the hypothesis that there is a significant main effect of ethanol and/or a significant interaction between ethanol and NMN. Differential ethanol effects, i.e., the effect of chronic ethanol differed between the group of animals exposed to NMN and the group of animals that were not, were also identified using a likelihood ratio statistic to test the hypothesis that there is a significant interaction between ethanol and NMN. Four groups of genes were identified: (1) NMN-dependent ethanol genes, (2) NMN-independent ethanol genes, (3) NMN-upregulated genes in control diet, and (4) NMN-downregulated genes in control diet. A false discovery rate (FDR) [7] was used to adjust for multiple testing.

### Functional enrichment analysis

Pathway analysis was performed with DAVID Bioinformatics Resources version 6.8 [27, 28]. A list of Ensembl IDs for all genes expressed above background in the liver, NMN-dependent ethanol genes, NMN-independent ethanol genes, NMN-upregulated genes in control diet, or NMN-downregulated genes in control diet were uploaded to the database. The list of genes expressed in the liver was used as the reference background. Enrichment analysis was performed using the KEGG pathway function to identify the most overrepresented pathway associated with our gene list. The significance of enrichment for each term/pathway was calculated using Fisher’s exact test (EASE score) to determine the probability that a given term was associated with more differentially expressed genes than expected by chance. The Benjamini-Hochberg procedure was used to adjust for multiple comparisons across terms. A threshold of significance for each term/pathway was set at nominal < 0.05.

### Immunoblotting

An aliquot of 20 μg of protein from mitochondrial, cytosolic, nuclear, or whole cell extracts was loaded onto a 12% stain-free SDS poly acrylamide gel. Gels were activated by the Chemidoc® MP (Bio-Rad, Hercules, CA, USA) before being transferred to PVDF membrane using a semi-dry transfer system (Bio-Rad Hercules, CA, USA), blocked using 5% (w/v) non-fat dry milk in Tris-buffered saline containing Tween 20 (0.1 % (v/v)) (TBST) for 1 h at room temperature and then incubated overnight with primary antibodies at 4 °C against Atf3 (ab207434) (Abcam, Cambridge, MA, USA), pErk1/2 (CS4370), Erk1/2 (CS4695), pP38 (CS9211), and P38 (9212) (Cell Signaling, Danvers, MA, USA). Membranes were washed three times by TBST, then incubated with appropriate secondary antibodies for 1 h at room temperature, and then washed three times with TBST. Clarity Western ECL Substrate (Bio-Rad Hercules, CA, USA) was applied before imaging via Chemidoc® MP (Bio-Rad, Hercules, CA, USA). A standard method using 2,2,2-trichloroethanol (Sigma, Saint Louis, MO, USA) stain was used to visualize overall protein load [32]. Densitometric analyses were performed using Imagelab 6.0 (Bio-Rad Hercules, CA). Blots were normalized to corresponding total protein. Densitometric figures and statistical analyses were performed by GraphPad Prism 6.04 (GraphPad, La Jolla, CA, USA). Figures represent mean +/− standard error of the mean (SEM, *n* > 3). Significance was calculated by two-way ANOVA with Tukey’s post hoc test. Results were considered significant if *p* < 0.05.

### Statistical analysis

As detailed above, statistical analyses and graphs were generated using Prism 6.04 (GraphPad, La Jolla, CA, USA). Graphics were generated using R and using Prism 6.04. Differences between groups were calculated using two-way ANOVA with Tukey’s post hoc test. Results were considered significant if *p* < 0.05.

## Supplementary information

**Additional file 1: Figure S1**: Assessment of liver triglycerides, liver to body weight ratio, and plasma ethanol concentrations. A) Ethanol consumption significantly increased liver triglycerides in saline and NMN groups. B) Ethanol slightly increased liver to body weight regardless of NMN treatment. C) Plasma ethanol levels significantly increased with ethanol feeding and NMN did not affect the blood ethanol concentrations. (n≥4) (mean +/- SEM) (*p<0.05) (**p<0.01) (***p<0.001) (****<0.0001). **Figure S2:** The effect of NMN in the control diet. A) A diagram showing the analysis performed on all protein coding genes to identify the effect of NMN in mice fed the control diet. B) Pathway analysis of upregulated genes by NMN in control diet. C) Pathway analysis of down regulated genes by NMN treatment in control diet. **Figure S3:** Western blot analysis demonstrates that NMN supplementation did not alter hepatic protein lysine acetylation globally within the mitochondria or specifically on SOD2 at lysine 68. Westerns were performed on the same blot, but were cropped for visualization. (mean +/-SEM) (****p<0.0001). **Figure S4:** Western blot analysis reveals that NMN supplementation did not alter protein lysine acetylation of the nuclear or cytosolic fractions of liver tissue. Saline and NMN Westerns were performed on the same blot, but were cropped for visualization. C, control; E, ethanol. (mean+/-SEM) (**p<0.01) (***p<0.001) (****p<0.0001).

**Additional file 2: Table S1**: Metabolomics.

**Additional file 3: Table S2**: RNA-seq.

## Data Availability

All data generated or analyzed during this study are included in this published article and its supplementary information files.

## Abbreviations

ALD: Alcoholic liver disease
ALT: Alanine aminotransferase
AST: Aspartate aminotransferase
Atf3: Activating transcription factor 3
CD38: Cluster of differentiation 38
CRE: cAMP response element
Dusp8: Dual specificity phosphatase 8
EDC: Ethanol-derived calories
Erk1/2: Extracellular signal-regulated kinase1/2
FDR: False discovery rate
Gadd45a: Growth arrest and DNA-damage-inducible 45 alpha
HILIC: Hydrophilic interaction chromatography
LPS: Lipopolysaccharide
MAPK: Mitogen-activated protein kinases
NMN: Nicotinamide mononucleotide
NMNAT1-3: Nicotinamide-nucleotide adenylyl transferase
NR: Nicotinamide riboside
PARP1: Poly(ADP-ribose) polymerase 1
SIRT1: Sirtuin 1
