# Supplementary material for: Investigating RNA expression profiles altered by nicotinamide mononucleotide therapy in a chronic model of alcoholic liver disease
Source: Hum Genomics. 2019 Dec 10;13:65. doi: 10.1186/s40246-019-0251-1 (PMC6902345; doi:10.1186/s40246-019-0251-1)

Supporting Information

Supporting Figure 1: Assessment of liver triglycerides, liver to body weight ratio, and plasma ethanol concentrations. A) Ethanol consumption significantly increased liver triglycerides in saline and NMN groups. B) Ethanol slightly increased liver to body weight regardless of NMN treatment. C) Plasma ethanol levels significantly increased with ethanol feeding and NMN did not affect the blood ethanol concentrations. ($n \geq 4$) (mean +/- SEM) (*$p<0.05$) (**$p<0.01$) (***$p<0.001$) (****<0.0001)

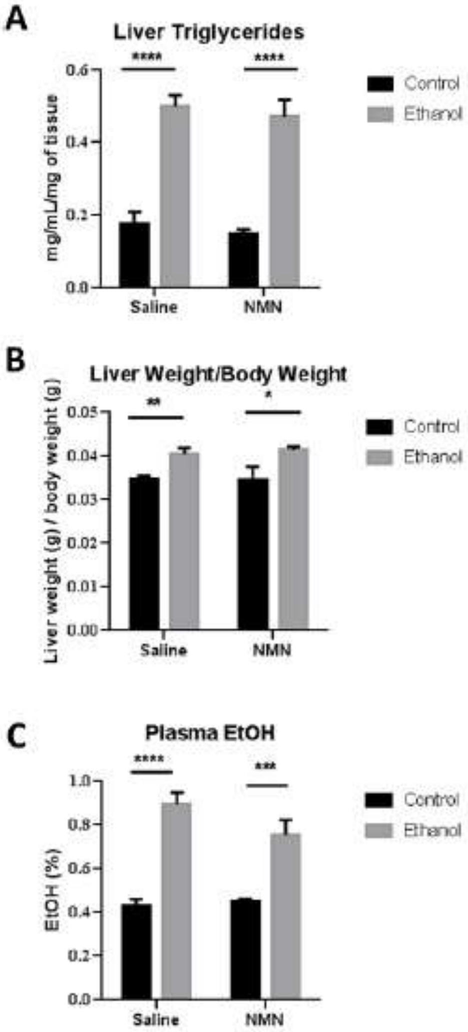

Supporting Figure 2: The effect of NMN in the control diet. A) A diagram showing the analysis performed on all protein coding genes to identify the effect of NMN in mice fed the control diet. B) Pathway analysis of upregulated genes by NMN in control diet. C) Pathway analysis of down regulated genes by NMN treatment in control diet.

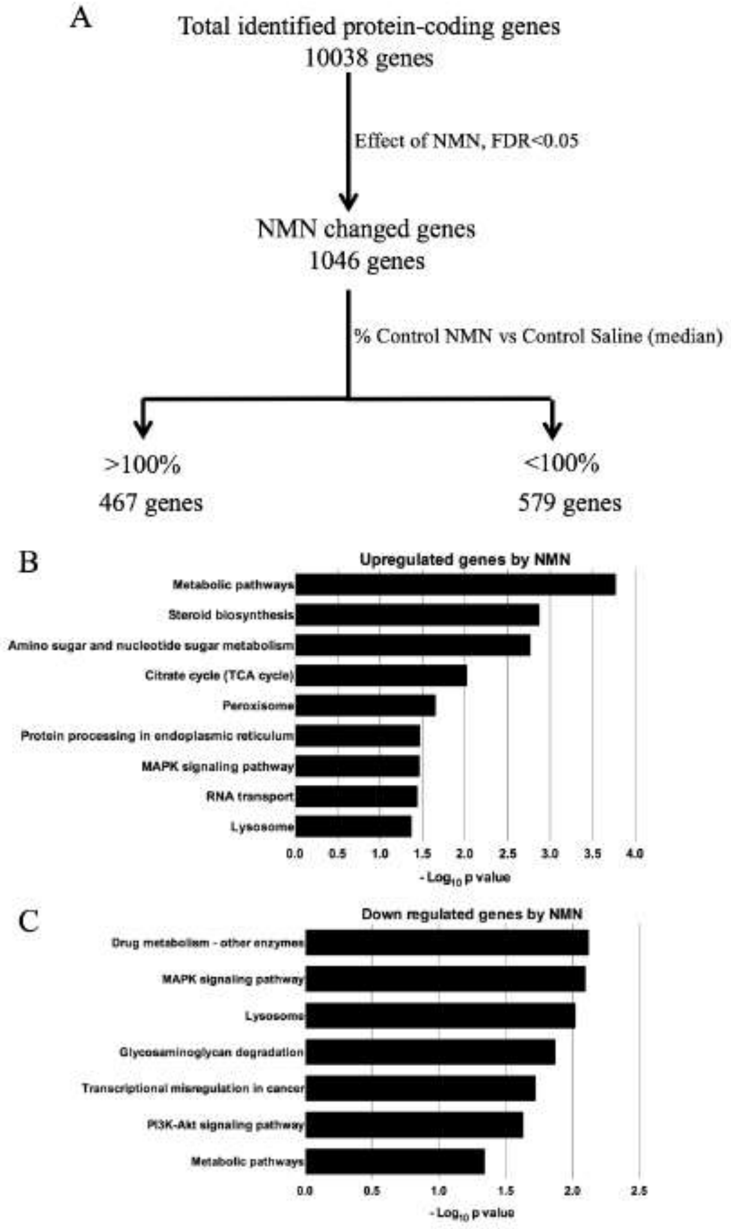

Supporting Figure 3: Western blot analysis demonstrates that NMN supplementation did not alter hepatic protein lysine acetylation globally within the mitochondria or specifically on SOD2 at lysine 68. Westerns were performed on the same blot, but were cropped for visualization. (mean +/-SEM) (****$p<0.0001$)

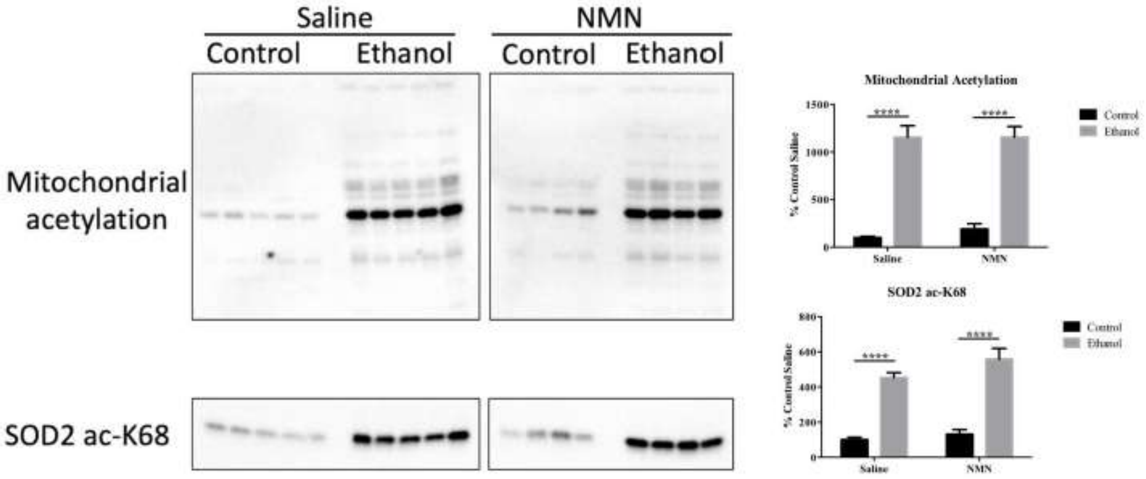

Supporting Figure 4: Western blot analysis reveals that NMN supplementation did not alter protein lysine acetylation of the nuclear or cytosolic fractions of liver tissue. Saline and NMN Westerns were performed on the same blot, but were cropped for visualization. C, control; E, ethanol. (mean+/-SEM) (**p<0.01) (***p<0.001) (****p<0.0001)

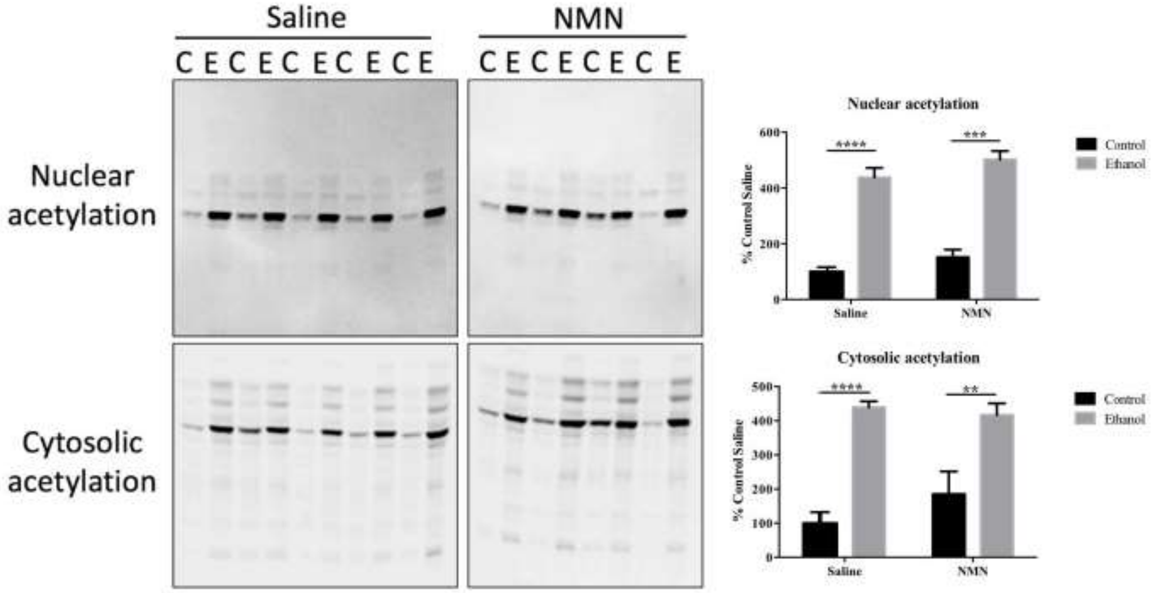

Supplement: Supplementary file 1 — Additional file 1: Figure S1: Assessment of liver triglycerides, liver to body weight ratio, and plasma ethanol concentrations. A) Ethanol consumption significantly increased liver triglycerides in saline and NMN groups. B) Ethanol slightly increased liver to body weight regardless of NMN treatment. C) Plasma ethanol levels significantly increased with ethanol feeding and NMN did not affect the blood ethanol concentrations. (n≥4) (mean +/- SEM) (*p<0.05) (**p<0.01) (***p<0.001) (****<0.0001). Figure S2: The effect of NMN in the control diet. A) A diagram showing the analysis performed on all protein coding genes to identify the effect of NMN in mice fed the control diet. B) Pathway analysis of upregulated genes by NMN in control diet. C) Pathway analysis of down regulated genes by NMN treatment in control diet. Figure S3: Western blot analysis demonstrates that NMN supplementation did not alter hepatic protein lysine acetylation globally within the mitochondria or specifically on SOD2 at lysine 68. Westerns were performed on the same blot, but were cropped for visualization. (mean +/-SEM) (****p<0.0001). Figure S4: Western blot analysis reveals that NMN supplementation did not alter protein lysine acetylation of the nuclear or cytosolic fractions of liver tissue. Saline and NMN Westerns were performed on the same blot, but were cropped for visualization. C, control; E, ethanol. (mean+/-SEM) (**p<0.01) (***p<0.001) (****p<0.0001). [file 40246_2019_251_MOESM1_ESM.pdf]
